# CNTNAP2 intracellular domain (CICD) generated by γ-secretase cleavage improves autism-related behaviors

**DOI:** 10.1038/s41392-023-01431-6

**Published:** 2023-06-05

**Authors:** Jing Zhang, Fang Cai, Renbin Lu, Xiaoliang Xing, Lu Xu, Kunyang Wu, Zishan Gong, Qing Zhang, Yun Zhang, Mengen Xing, Weihong Song, Jia-Da Li

**Affiliations:** 1 Furong Laboratory, Center for Medical Genetics, Hunan Key Laboratory of Animal Models for Human Diseases, Hunan Key Laboratory of Medical Genetics, Hunan International Scientific and Technological Cooperation Base of Animal Models for Human Diseases, School of Life Sciences, Central South University, Changsha, 410078 Hunan China; 2 Oujiang Laboratory (Zhejiang Lab for Regenerative Medicine, Vision and Brain Health), Wenzhou, Zhejiang 325000 China; 3 Townsend Family Laboratories, Department of Psychiatry, The University of British Columbia, 2255 Wesbrook Mall, Vancouver, BC V6T 1Z3 Canada; 4 Institute of Aging, Key Laboratory of Alzheimer’s Disease of Zhejiang Province, School of Mental Health and Kangning Hospital, The Second Affiliated Hospital and Yuying Children’s Hospital, Wenzhou Medical University, Wenzhou, Zhejiang 325035 China; 5 Advanced Innovation Center for Human Brain Protection, The National Clinical Research Center for Geriatric Disease, Xuanwu Hospital, Capital Medical University, Beijing, 100053 China

**Keywords:** Neurodevelopmental disorders, Molecular biology

## Abstract

As the most prevalent neurodevelopmental disorders in children, autism spectrum disorders (ASD) are characterized by deficits in language development, social interaction, and repetitive behaviors or inflexible interests. *Contactin associated protein like 2 (CNTNAP2)*, encoding a single transmembrane protein (CNTNAP2) with 1331 amino acid residues, is a widely validated ASD-susceptible gene. *Cntnap2*-deficient mice also show core autism-relevant behaviors, including the social deficits and repetitive behavior. However, the cellular mechanisms underlying dysfunction CNTNAP2 and ASD remain elusive. In this study, we found a motif within the transmembrane domain of CNTNAP2 was highly homologous to the γ-secretase cleavage site of amyloid-β precursor protein (APP), suggesting that CNTNAP2 may undergo proteolytic cleavage. Further biochemical analysis indicated that CNTNAP2 is cleaved by γ-secretase to produce the CNTNAP2 intracellular domain (CICD). Virally delivery of CICD to the medial prefrontal cortex (mPFC) in *Cntnap2*-deficient (*Cntnap2*^*−/−*^) mice normalized the deficit in the ASD-related behaviors, including social deficit and repetitive behaviors. Furthermore, CICD promoted the nuclear translocation of calcium/calmodulin-dependent serine protein kinase (CASK) to regulate the transcription of genes, such as Prader Willi syndrome gene *Necdin*. Whereas *Necdin* deficiency led to reduced social interaction in mice, virally expression of *Necdin* in the mPFC normalized the deficit in social preference of *Cntnap2*^*−/−*^ mice. Our results thus reveal a critical function of CICD and highlight a role of the CNTNAP2-CASK-Necdin signaling pathway in ASD.

## Introduction

Autism spectrum disorders (ASD) are the most prevalent neurodevelopmental disorders in children, with a group of heterogeneous clinical syndromes, characterized by deficits in language development, social interaction, and repetitive behaviors or inflexible interests.^1^ Over 70% ASD patients are accompanied with comorbidities, such as attention-deficit hyperactivity disorder (ADHD), epilepsy, anxiety, depression, sleep disturbance, gastrointestinal and immune problems.^2,3^ In recent decades, the incidence of ASD is steadily increasing up to 1/44 according to the report from the Center for Disease Control and Prevention (CDC) in 2021. However, the high heterogeneity in the clinical and genetic features of ASD greatly hampered the understanding of their genetic and pathological mechanisms.^4,5^

ASD is highly heritable disorder, and several hundred genes are associated with increased risk of developing ASD.^6,7^ The findings from genetics, neuropathology and therapeutics disciplines converge attentions on several potential common molecular or cellular pathways, including PI3K/mTOR signaling pathways, oxytocinergic activities and defective synaptic functioning.^2^ PI3K/mTOR signaling pathways contribute to synaptic protein synthesis and thereby altering synaptic functions, and closely linked to activation of some neuron surface receptors, such as N-Methyl-D-aspartic acid-type glutamate receptors (NMDARs), metabotropic glutamate receptors (mGluRs), and AMPA-type glutamate receptors (AMPARs).^8,9^ Moreover, mTOR inhibitors such as rapamycin and everolimus can ameliorate behavioral deficits in certain autistic animal models.^10,11^ Genetic variants in oxytocin (OXT) and oxytocin receptor (OXTR) have been reported to be linked to autism, and *Oxt* or *Oxtr* knockout mice exhibit ASD-associated behavioral abnormalities.^12–15^ As a neurotransmitter, dynamic alterations in OXT concentration and its receptors density may influence the neural circuit and thus affect the behavioral performance.^14,16,17^ Some typical animal models of ASD also have abnormal oxytocin system, and OXT treatment can effectively rescue the social deficit.^18^ Probable explanation is that OXT from the paraventricular nucleus (PVN) enhances the dopamine release in the ventral tegmental area (VTA), and ultimately activates the excitability of dopamine D1 neurons in the nucleus accumbens (NAc) to enhance social behavior.^19^ Recent studies have also highlighted the significance of synapse formation and stabilization, synaptic modification and functional connection establishment processes in ASD etiology.^20^ Abnormalities in synaptic proteins involved in cell adhesion, scaffolding, or signaling may underlie the etiology of ASD.^21^ For instance, neurexins and neuroligins are presynaptic and postsynaptic binding partners to mediate synapse formation and stabilization. In humans, variants in genes encoding neuroligins or neurexins have been associated with ASD.^22^

*Contactin associated protein like 2 (CNTNAP2)*, a widely validated ASD risk gene,^23–26^ encodes CNTNAP2 protein with 1331 amino acid residues. CNTNAP2 is a single transmembrane protein of Neurexin superfamily, localized in the post-synaptic region with a large extracellular domain and a short intracellular domain.^27^ *Cntnap2*-deficient mice show core autism-relevant behaviors, including the social deficits and repetitive behavior.^28^ Functionally, CNTNAP2 is involved in the growth of synaptic spines, neuronal migration and neuronal network activity.^29,30^ RNAi-mediated knockdown of Cntnap2 globally decreases the dendritic arborization as well as spine development, resulting in a suppression of neural network activity. And loss of Cntnap2 in neurons causes reduction in interneurons number, and alterations in neuronal migration and cortical layer patterning. Furthermore, it is reported that repression of Cntnap2 expression in the prefrontal cortex leads to a decrease in the number of functional synapses and an imbalance of E/I balance.^31^ Nevertheless, the underlying cellular mechanisms are still unclear.

Previously, we identified a panel of differentially expressed genes in the hippocampus of *Cntnap2*^*−/−*^ mice;^32^ however, how CNTNAP2 regulates the expression of these genes is unknown. Numerous single transmembrane proteins undergo two steps of proteolytic processing. The first cleavage at the extracellular juxtamembrane region releases a soluble extracellular fragment, and the second cleavage by γ-secretase within the transmembrane region produces an intracellular domain.^33^ For instance, Notch receptors undergo two sequential proteolytic cleavages upon ligand binding and release the Notch intracellular domain (NICD), which translocates to the nucleus and activates its target genes by forming a complex with transcription factor CBF1/Su(H)/Lag-1 or CSL.^34–36^ Furthermore, the amyloid-β precursor protein (APP) undergoes successive cleavage by β- and γ-secretases to generate the amyloid β protein (Aβ), the secreted APP (sAPP), and APP intracellular domain (AICD).^37,38^ AICD could translocate into the nucleus to regulate gene expression by forming a complex with Fe65 and Tip60.^39^

In this study, we found that a motif within the transmembrane domain of CNTNAP2 was highly homologous to APP’s γ-secretase cleavage site, suggesting that CNTNAP2 may undergo proteolytic cleavage. Our biochemical studies demonstrated that CNTNAP2 was first cleaved in extracellular juxtamembrane domain releasing a soluble extracellular fragment and a membrane-tethered 20KDa C-terminal fragment (CTF). The CTF was further processed by γ-secretase to generate the CNTNAP2 intracellular C-terminal domain (CICD). Overexpression of CICD in the mPFC of *Cntnap2*^*−/−*^ mice rescued both social deficit and repetitive behaviors. We further found that CICD promotes the nuclear entry of calcium/calmodulin-dependent serine protein kinase (CASK) to regulate the expression of a panel of target genes. The Prader Willi gene *Necdin* is one of such target genes and partly responsible for the deficit of autism-related behaviors in *Cntnap2*^*−/−*^ mice, as overexpression of *Necdin* in the mPFC of *Cntnap2*^*−/−*^ mice normalized the social deficit.

## Results

### CNTNAP2 undergoes proteolytic cleavage

**Fig. 1:**
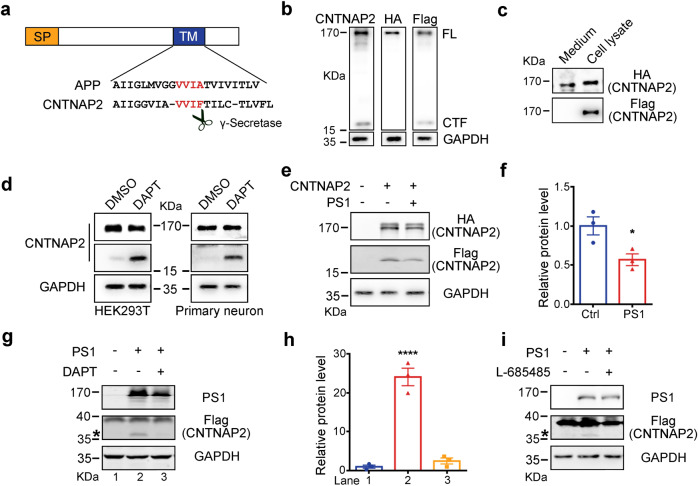
CNTNAP2 undergoes proteolytic cleavage. **a** Amino acid sequence alignment of the transmembrane domains of CNTNAP2 and APP. A potential recognition site of γ-secretase is highlighted in red. SP, signal peptide; TM, transmembrane region. **b** Identification of a ~20 KDa fragment (CTF) in the cell lysates from HEK293T cells transfected with pRK5-HA-CNTNAP2-Flag plasmid with antibodies against CNTNAP2 and Flag, but not the antibody against HA. FL, full length CNTNAP2. **c** In the conditioned medium from HEK293T cells transfected with pRK5-HA-CNTNAP2-Flag plasmid, a fragment of slight smaller than full length CNTNAP2 was detected with an antibody against HA, but not an antibody against Flag. However, both antibodies against HA and Flag were able to detect the full length CNTNAP2 in the cell lysates from HEK293T cells transfected with pRK5-HA-CNTNAP2-Flag plasmid. **d** Treatment with a γ-secretase inhibitor DAPT (10 μM) increased the CTF in the cell lysates from HEK293T cells transfected with pRK5-HA-CNTNAP2-Flag plasmid (left) or 14 days-cultured primary hippocampal neurons (right). **e**, **f** The pRK5-HA-CNTNAP2-Flag plasmid was transfected alone or with PS1-expressing plasmid into MEF cells taken from mice deficient in both PS1 and PS2 (NN cells), the CTF in the cell lysates was significantly decreased in the presence of PS1, *n* = 3, *p < 0.05, unpaired *t*-test. The pRK5-HA-CNTNAP2-GFP-Flag plasmid was transfected alone or with PS1-expressing plasmid into NN cells in the presence or absence of a γ-secretase inhibitor DAPT (10 μM), the product of γ-secretase was marked with a star (*) in (**g**) and quantified in (**h**). *n* = 3;****p < 0.0001, unpaired *t*-test. **i** The pRK5-HA-CNTNAP2-GFP-Flag plasmid was transfected alone or with PS1-expressing plasmid into NN cells in the presence or absence of a γ-secretase inhibitor L-685485, the product of γ-secretase was marked with a star (*)

Proteolytic processing of many single transmembrane proteins such as Notch and APP^34,36,39–41^ to release the intracellular domain is required for their functions. We found that CNTNAP2 contains a VVIF motif within its transmembrane domain that is highly homologous to the VVIA of APP’s γ-secretase cleavage site (Fig. 1a). To investigate whether CNTNAP2 undergoes proteolytic cleavage, plasmid pRK5-HA-CNTNAP2-Flag (CNTNAP2 with an HA tag after signal peptide (SP) and a Flag tag at the C-terminus; Supplementary Fig. 1a, upper) was transfected into HEK293T cells, CNTNAP2 was expressed on the cell surface (Supplementary Fig. 1b). Western blot with the cell lysates showed a full length CNTNAP2 of ~170KDa as detected by the antibodies against CNTNAP2, C-terminal Flag tag or N-terminal HA tag (Fig. 1b). A band of ~20 KDa was also detected by the antibodies against CNTNAP2 or C-terminal Flag tag, but not by the antibody against N-terminal HA tag (Fig. 1b). We also detected the ~20 kDa fragment in a variety of brain regions by using the CNTNAP2 antibody (Supplementary Fig. 2a). The ~20 KDa fragment was immunoprecipitated by a Flag antibody and subjected to mass spectrometry (MS) analysis (Supplementary Fig. 2b). As shown in Supplementary Fig. 2c, multiple peptide fragments corresponding to the CNTNAP2 C-terminus were identified. Furthermore, a protein with a molecular weight slightly less than the full length CNTNAP2 was also detected in the culture medium by the antibody against HA tag but not by the antibody against the Flag tag (Fig. 1c). These data indicated that CNTNAP2 may undergo proteolytic cleavage, producing a soluble extracellular domain and a membrane-tethered 20 KDa C-terminal fragment (CTF).

### The γ-secretase cleaves CNTNAP2 to generate CICD

CNTNAP2 contains a potential γ-secretase cleavage site (Fig. 1a). As γ-secretase is involved in the proteolytic cleavage of type I transmembrane proteins such as APP and Notch receptors,^36,40–42^ we then sought to investigate the roles of γ-secretase in the proteolytic processing of CNTNAP2. To this end, DAPT, a γ-secretase inhibitor, was added to the HEK293T cells transfected with pRK5-HA-CNTNAP2-Flag. DAPT treatment significantly increased the level of ~20 KDa CTF (Fig. 1d, left). The CTF of endogenous CNTNAP2 proteolytic product was also increased in the primary hippocampal neurons after DAPT treatment (Fig. 1d, right). These data suggest that the CTF is a potential substrate of γ-secretase.

Presenilins (PS1 and PS2) are the catalytic subunits of the γ-secretase complex, and knockout (KO) of PS1 and PS2 abolished γ-secretase activity.^42^ To further investigate the function of γ-secretase in the processing of CNTNAP2, PS1-expressing plasmid and pRK5-HA-CNTNAP2-Flag were transfected into PS1/2-double-KO MEF cells (NN cells).^37^ As shown in Figs. 1e, f, expression of PS1 significantly decreased the ~20KDa CTF level in the presenilins-deficient NN cells, further implicating the CTF as a substrate for γ-secretase.

We speculate that the fragment after γ-secretase processing is too small to be detected. Therefore, plasmid pRK5-CNTNAP2-GFP-Flag expressing EGFP between the Flag tag and the C-terminus of CNTNAP2 (Supplementary Fig. 1a, lower) was transfected into the NN cells, and a band of ~40 KDa corresponding to the EGFP-tagged CTF was detected. However, co-transfection of the PS1 generated a ~35 KDa fragment, which was abolished after treatment with DAPT or L-685485, two γ-secretase inhibitors (Fig. 1g–i). Further analyses by N-terminal sequencing and mass spectrum showed the N-terminal sequence of the cleaved C-terminal fragment as LVFLIR, corresponding to the ε-site of APP by γ-secretase cleavages^43^ (Supplementary Fig. 3). These results clearly demonstrated that the ~35KDa fragment is an EGFP-tagged CNTNAP2 intracellular domain (CICD) generated by the γ-secretase cleavage.

### CICD rescues autism-related behaviors in *Cntnap2*^*−/−*^ mice

**Fig. 2:**
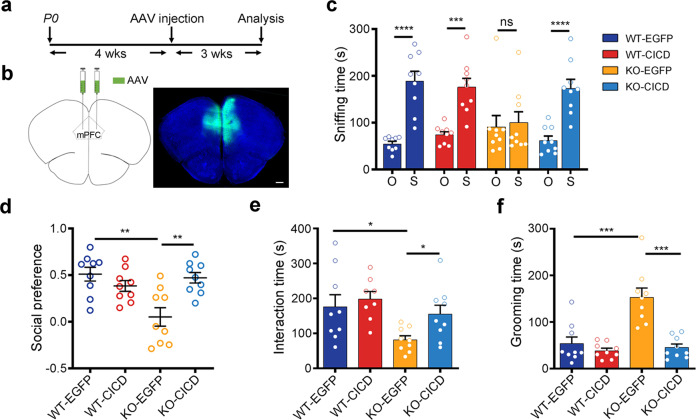
Viral delivery of CICD to the mPFC rescued autism-related deficits in *Cntnap2*^*−/−*^ mice. **a** The schematic diagram for the experimental procedure. P0, postnatal day 0. **b** The schematic representation of virus injection in the mPFC (left) and a representative EGFP immunofluorescence signal in the mPFC (right). Scale bar, 500 µm. **c** The sniffing time of test mice spent on an object (O) or a stranger mouse (S) as measured with three-chamber test. **d** The social preference index in the three-chamber test was calculated from (**c**). **e** The time of test mice spent on social interaction as measured in reciprocal interaction test. **f** The time of test mice spent on self-grooming. Data were mean ± SEM, hollow circles represented individual mice, *n* = 9. *p < 0.05, **p < 0.01, ***p < 0.001, ****p < 0.0001, unpaired *t*-test

*Cntnap2*^*−/−*^ mice showed core autism-relevant deficiency, including impaired social interaction and repetitive behaviors.^28^ To investigate the role of CICD in the ASD-relevant behaviors, recombination adeno-associated virus expressing CICD (rAAV-EF1a-CICD-2A-EGFP, AAV-CICD) or EGFP control (rAAV-EF1a-2A-EGFP, AAV-EGFP) (Supplementary Fig. 4a) was bilaterally infused into the mPFC of 4-week-old male WT or *Cntnap2*^*−/−*^ mice, and behavioral tests were performed at 3 weeks after injection (Fig. 2a, b). The immunofluorescent signal of EGFP revealed the infection of AAV into neurons but not astrocytes and microglia (Supplementary Fig. 4b, c).

Animals were habituated in a three-chamber test (Supplementary Fig. 5) and the sociability test showed that while WT mice preferred to interact with the stranger mouse (S) than the object (O), *Cntnap2*^*−/−*^ mice injected with AAV-EGFP did not differentiate the object and stranger mouse (Fig. 2c, d), consistent with previous reports.^28^ However, *Cntnap2*^*−/−*^ mice injected with AAV-CICD in the mPFC spent significantly more time with the mouse than the object, and the social preference was comparable to that of WT mice (Fig. 2c, d).

A reciprocal social interaction test was also performed. *Cntnap2*^*−/−*^ mice injected with AAV-EGFP spent significantly less time on reciprocal social interaction than WT mice (Fig. 2e). However, viral delivery of CICD to the mPFC improved the sociability in *Cntnap2*^*−/−*^ mice and increased their reciprocal social interaction time to the comparable levels of WT mice (Fig. 2e).

Repetitive behavior is one of the core clinic symptoms in ASD.^44^ Consistent with previous reports,^28^ *Cntnap2*^*−/−*^ mice injected with AAV-EGFP spent significantly longer time on grooming themselves as compared with the WT controls (Fig. 2f). Viral delivery of CICD to the mPFC significantly decreased the self-grooming behaviors in *Cntnap2*^*−/−*^ mice to the level of WT controls (Fig. 2f). Taken together, our results indicated that overexpression of CICD in the mPFC rescued both social deficit and repetitive behaviors, suggesting a critical role of CICD in the autism-related behaviors.

### CICD transcriptionally activates *Necdin* expression

**Fig. 3:**
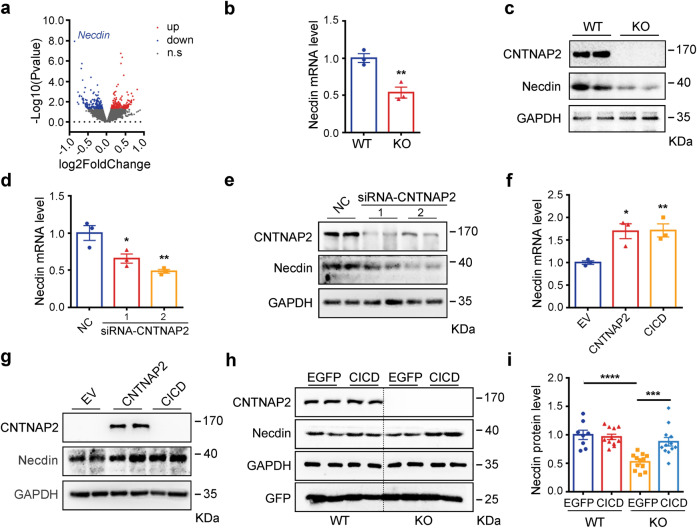
CICD regulates the transcription of Prader Willi gene *Necdin*. **a** A volcanic map of differentially expressed genes in the hippocampus from wild-type (WT) and *Cntnap2*^*−/−*^ as assayed with RNA-seq. *Necdin* was the most down-regulated gene. **b, c** The relative levels of *Necdin* in the mPFC of WT and *Cntnap2*^*−/−*^ mice were measured with qPCR (*n* = 3) and Western blot, respectively. RNA interference of *Cntnap2* in N2a cells led to reduction of mRNA and protein levels of *Necdin* as measured with qPCR (**d**, *n* = 3) and Western blot (**e**), respectively. Overexpression of *Cntnap2* and CICD in N2a cells increased the mRNA and protein levels of Necdin as measured with qPCR (**f**, n = 3) and Western blot (**g**), respectively. **h, i** Overexpression of CICD by AAV normalized the Necdin protein levels in the mPFC of *Cntnap2*^*−/−*^ mice. Statistics data from WT-EGFP mice (*n* = 8), WT-CICD mice (*n* = 11), KO-EGFP and KO-CICD mice (*n* = 12) were shown in (**i**). Data were mean ± SEM, *p < 0.05, **p < 0.01,***p < 0.001, ****p < 0.0001, unpaired *t*-test

Some single transmembrane proteins, such as APP and Notch receptors, are proteolytically cleaved to produce intracellular domains, which translocate into the nucleus to transcriptionally regulate gene expression.^34,36,39^ Previously by performing RNA-sequencing analysis, 99 significantly down-regulated genes and 90 significantly up-regulated genes were identified in the hippocampus of *Cntnap2*^*−/−*^ mice.^32^ Among these genes, *Necdin* in the Prader-Willi syndrome (PWS) chromosomal region is the most notably changed gene (Fig. 3a), and patients with PWS also present with autism-related symptoms.^45^ Furthermore, *Necdin* was considered as a driver gene contributing to the ASD-like phenotypes in a mouse model of paternal 15q duplications.^46^

*Necdin* expression in the *Cntnap2*^-/-^ mice was also significantly decreased at mRNA as well as protein levels in the mPFC (Fig. 3b, c; Supplementary Fig. 6a). Further, knockdown of *Cntnap2* with siRNAs in N2a neuronal cells significantly downregulated *Necdin* (Fig. 3d, e; Supplementary Fig. 6b), whereas overexpression of CNTNAP2 increased the endogenous *Necdin* level (Fig. 3f, g). Interestingly, overexpression of CICD in N2a cells also significantly upregulated the mRNA and protein levels of endogenous *Necdin* (Fig.3f, g; Supplementary Fig. 6c). Consistently, viral injection of CICD to the mPFC of *Cntnap2*^-/-^ mice also normalized the Necdin protein to the level of WT mice (Fig. 3h, i). Our results indicated that *Necdin* is a downstream target gene of CNTNAP2 and CICD mediates the effect of CNTNAP2 on activating the *Necdin* gene expression.

### Necdin alleviates social deficit in *Cntnap2*^*−/−*^ mice

**Fig. 4:**
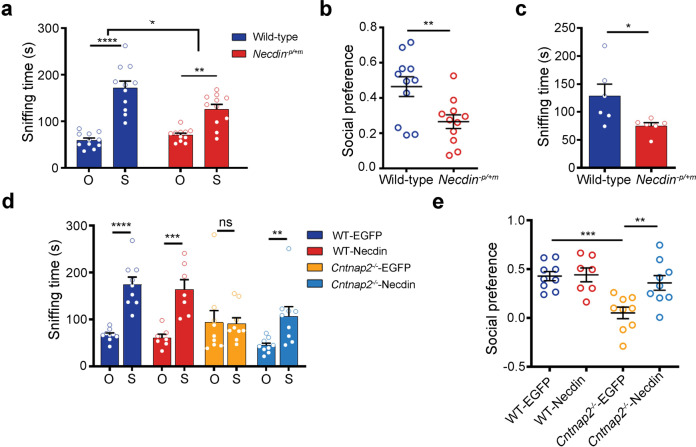
Necdin alleviates social deficit in *Cntnap2*^*−/−*^ mice. **a** *Necdin*-deficient (*Necdin*^-p/+m^) mice showed reduced sociability in three-chamber test. The sniffing time of test mice spent on an object (O) or a mouse (S) was measured, *n* = 11 mice/genotype. **b** The social preference in the three-chamber test of *Necdin*^*−p/+m*^ mice were significantly lower than WT mice, *n* = 11 mice/genotype. **c** The time of test mice spent on social interaction as measured in a reciprocal interaction test, *n* = 6 mice/genotype. **d** WT and *Cntnap2*^*−/−*^ mice were injected with control virus (EGFP) or Necdin virus bilaterally into the mPFC at the age of 4 weeks, and sociability was measured with three-chamber test at 3 weeks after injection. The sniffing time of test mice spent on an object (O) or a mouse (S) was measured, *n* = 9 mice/group. **e** The social preference of *Cntnap2*^*−/−*^ mice was improved after injection of AAV-Necdin in the mPFC, *n* = 9 mice/group. Data were mean ± SEM, hollow circles represented individual mice. *p < 0.05, **p < 0.01, ***p < 0.001, ****p < 0.0001. A Bonferroni’s multiple comparisons test was used in (**a**), and an unpaired *t*-test was used in (**b**, **e**)

To determine the effects of Necdin on autism-related phenotypes, animals were habituated in a three-chamber test (Supplementary Fig. 7a, b) and then subjected to the sociability test. Both *Necdin*-deficient (*Necdin*^-p/+m^)^47^ and WT mice preferred to interact with the stranger mouse (S) than the object (O) (Fig. 4a); however, *Necdin*^-p/+m^ mice spent significantly less time on interacting with a mouse and the preference index of *Necdin*^-p/+m^ mice was significantly lower than WT mice (Fig. 4a, b). The reciprocal social interaction test further confirmed the reduced sociability in *Necdin*^-p/+m^ mice (Fig. 4c). Nevertheless, no significant genotypic difference was found in the grooming test (Supplementary Fig. 7c).

In order to determine if *Necdin* deficiency is responsible for the autism-related deficits in *Cntnap2*^*−/−*^ mice, AAV-EGFP or rAAV-EF1a-Necdin-2A-EGFP (AAV-Necdin) were injected into the mPFC of WT and *Cntnap2*^-/-^ mice at the age of 4 weeks and the social behaviors and grooming were measured at the age of 7 weeks (Supplementary Fig. 8a). In the three-chamber test, animals were habituated (Supplementary Fig. 8b) and then subjected to the sociability tests. As shown in Fig. 4d and Supplementary Fig. 8c, overexpression of *Necdin* in the mPFC normalized the social deficits in *Cntnap2*^*−/−*^ mice. Meanwhile, the social preference was also improved significantly (Fig. 4e). However, there was no effect on the repetitive behavior as measured with a grooming test (Supplementary Fig. 8d).

### CASK regulates the transcription of *Necdin*

**Fig. 5:**
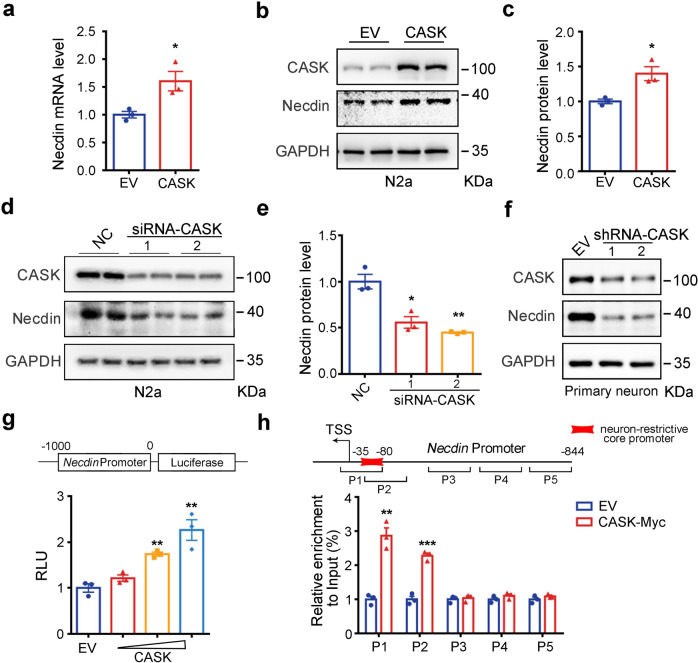
CASK promotes the transcription of *Necdin*. **a–c** Overexpression of CASK in N2a cells increased the mRNA and protein levels of *Necdin* as measured with qPCR and Western blot. **d, e** RNA interference of CASK in N2a cells decreased Necdin protein levels. Representative immunoblots and statistics data of three independent experiments were shown. **f** RNA interference of CASK by lentivirus leads to decreased Necdin protein levels in primary neurons. **g** Overexpression of CASK promoted the *Necdin* promoter activity in a dose-dependent manner. A reporter plasmid containing luciferase gene under the control of *Necdin* promoter and different doses of CASK expression plasmids or empty vector were transfected into HEK293T cells, the luciferase activity was measured. RLU, relative light unit. **h** N2a cells transfected with pRK5 or pRK5-CASK-Myc were subjected to chromatin immunoprecipitation (ChIP) by using an antibody against Myc. The precipitated DNA was amplified with five pairs primers targeting different regions of *Necdin* promoter (above). The qPCR products from primer pairs 1 and 2 were significantly higher in cells transfected with pRK5-CASK-Myc than cells transfected with pRK5. EV, empty vector. Data were mean ± SEM, *n* = 3, *p < 0.05, **p < 0.01, ***p < 0.001; unpaired *t*-test

To understand how CICD regulates gene expression, we searched if any CNTNAP2-interactive proteins have regulatory function in transcription. To this end, CASK stands out as an interesting candidate. CASK, a membrane-anchored ASD-risk protein, translocates into the nucleus and interacts with transcription factors to regulate gene transcription.^48,49^ The interaction between CASK and CICD was confirmed by using a co-immunoprecipitation assay (Supplementary Fig. 9a). We then investigated the effect of CASK on *Necdin* gene expression. We found that overexpression of CASK increased the mRNA and protein levels of *Necdin* in N2a cells (Fig. 5a–c), and RNA interference of CASK decreased the expression of *Necdin* in N2a cells as well as in primary neurons (Fig. 5d–f).

A luciferase reporter plasmid containing *Necdin* promoter (−1 kb to TSS) was generated for transcriptional activation assay. Overexpression of CASK dose-dependently promoted the *Necdin* promoter activity (Fig. 5g, Supplementary Fig. 9b). Furthermore, N2a cells were transfected with pRK5-CASK-Myc, and CASK-bound DNA was immunoprecipitated by using a Myc antibody. Five pairs of primers, spanning the −844 bp to TSS of *Necdin* promoter, were used to amplify the CASK-bound DNA by using quantitative PCR (qPCR). As shown in Fig. 5h and Supplementary Fig. 9c, DNA fragments including the neuron-restrictive core promoter region of *Necdin* (−80 to −35 bp) were significantly enriched in cells expressing CASK-Myc.

### CICD regulates *Necdin* expression by facilitating nuclear translocation of CASK

**Fig. 6:**
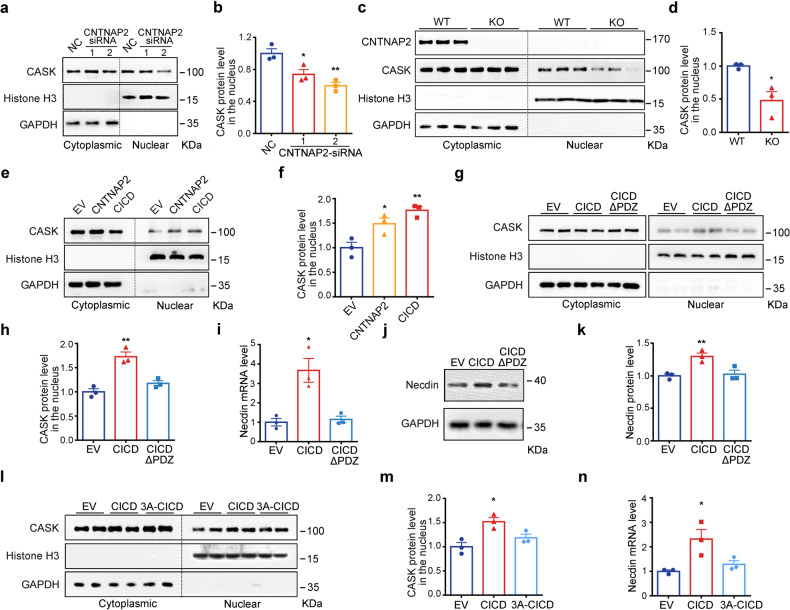
CICD regulates transcription by facilitating nuclear translocation of CASK. **a**, **b** RNA interference of CNTNAP2 in N2a cells led to decreased nuclear distribution of CASK. **c, d** The nuclear form of CASK was significantly reduced in the mPFC from *Cntnap2*^*−/−*^ mice. **e, f** Overexpression of CNTNAP2 or CICD in N2a cells increased the nuclear distribution of CASK. **g, h** Overexpression of CICDΔPDZ failed to alter the cyto-nuclear distribution of CASK in N2a cells. **i–k** Overexpression of CICDΔPDZ in N2a cells failed to upregulate the mRNA and protein levels of Necdin. **l–n** Overexpression of 3A-CICD failed to alter the cyto-nuclear distribution of CASK in N2a cells and Necdin mRNA level . Data were mean ± SEM, *n* = 3, *p < 0.05, **p < 0.01, unpaired *t*-test

As a membrane-anchored protein, CASK can also shuttle into the nucleus to regulate gene expression.^48^ We then sought to investigate if CICD influence the nuclear distribution of CASK. To this end, the endogenous CASK in the cytoplasmic and nuclear fractions from N2a cells or mPFC were measured. As shown in Fig. 6a, b, RNA interference of *Cntnap2* significantly inhibited the nuclear entry of CASK in N2a cells. Furthermore, the endogenous CASK in nucleus was also significantly decreased in the mPFC of *Cntnap2*^*−/−*^ mice than WT mice (Fig. 6c, d). Conversely, the nuclear form of CASK was significantly increased after overexpression of full length CNTNAP2 or CICD (Fig. 6e, f).

As CASK binds to the PDZ-binding motif at the C-terminus of CNTNAP2,^50^ we therefore generated a mutant CICD without this PDZ-binding motif (CICDΔPDZ), which did not interact with CASK in the co-immunoprecipitation assay (Supplementary Fig. 9a). As shown in Fig. 6g, h, CICDΔPDZ failed to change the nuclear-cytoplasmic distribution of CASK. Consistently, CICDΔPDZ was unable to upregulate the *Necdin* expression as the full length CICD (Fig. 6i–k). A triplet of basic amino acids (RHK) in the N-terminus of CICD may serve as a potential nuclear localization signal (NLS) (Supplementary Fig. 10a). Therefore, we mutated RHK to AAA in CICD (namely 3A-CICD). 3A-CICD failed to promote the nuclear localization of CASK and subsequent up-regulation of *Necdin* expression (Fig. 6l–n; Supplementary Fig. 10b-c).

Immunofluorescence was also carried out to see the effect of CICD and its mutants on the subcellular localization of CASK. CASK was detected in the cytoplasm and peri-membrane when it was expressed alone in N2a cells. CASK was clearly identified in the nucleus when it was co-expressed with CICD. However, no CASK was detected in the nucleus when it was co-expressed with CICDΔPDZ or 3A-CICD. On the other hand, CICD and CICDΔPDZ were detected in both cytoplasm and nucleus, whereas 3A-CICD was only detected in the cytoplasm, indicating an important role for the triplet of basic amino acids (RHK) in the nuclear localization (Supplementary Fig. 10d).

## Discussion

**Fig. 7:**
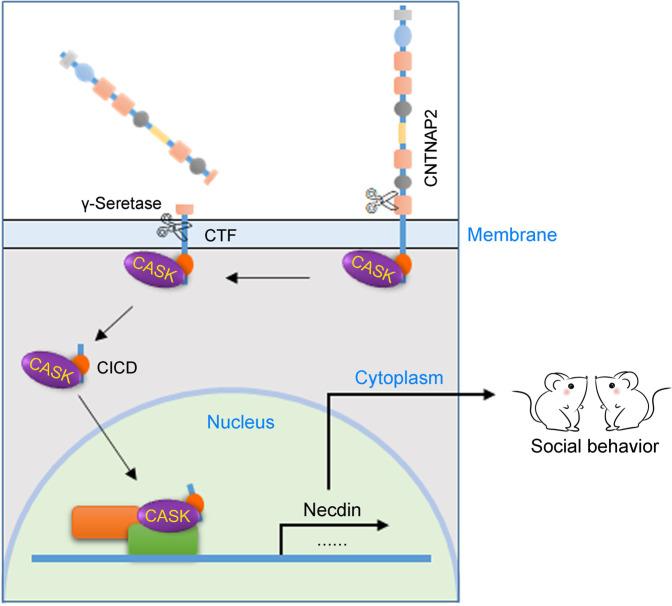
Schematic presentation of a proposed mechanism underlying the function of CICD generated by γ-secretase cleavage in the autism-related behaviors. CNTNAP2 is initially processed at the extracellular region to generate the soluble extracellular domain and a membrane-tethered CTF fragment, which was further cleaved by the γ-secretase to generate CICD. CICD then functions by promoting the nuclear distribution of CASK to regulate the transcription of genes such as *Necdin*, which promotes the sociability

Our study revealed a critical role for CICD generated by γ-secretase cleavage of CNTNAP2 in the autism-related behaviors, as overexpression of CICD in the mPFC of *Cntnap2*^*−/*−^ mice fully rescues the social deficit as well as repetitive behaviors. Mechanistically, CICD may function by promoting the nuclear distribution of CASK to regulate gene expression. Our study implicates three ASD-susceptible genes, *CNTNAP2*, *Necdin* and *CASK*, may participate in the autism-related behaviors through a common pathway (Fig. 7). Further, as many CASK-interactive proteins, such as neurexin, are also type I transmembrane proteins and involved in ASD;^51^ it will be intriguing to investigate if the pathway uncovered in this study is also related to the etiology of ASD induced by deletion/mutation of those genes.

Our data indicate that CNTNAP2 was initially processed at the extracellular region to generate the soluble extracellular domain and a membrane-tethered CTF fragment (~20KDa), which was further cleaved by the γ-secretase to generate CICD (Fig. 7). Recently, Martin-de-Saavedra et al. reported a similar finding. They found that CNTNAP2 underwent activity-dependent ectodomain shedding and released a soluble extracellular fragment (CNTNAP2 ectodomain), which is involved in the regulation of Ca^2+^ homeostasis and network synchrony.^52^ While our data indicate that CICD is largely, if not all, responsible for the autism-related behaviors, the CNTNAP2 ectodomain may underlie the epilepsy in *Cntnap2*^*−/*−^ mice. These two complementary studies uncover that CNTNAP2 exert diverse functions through different proteolytic products.

CNTNAP2 ectodomain is detectable in human cerebrospinal fluid (CSF), and its level is reduced in the CSF of individuals with ASD.^52^ It deserves to investigate if and how ASD-related *CNTNAP2* mutations affect the proteolytic processing of CNTNAP2 protein. We speculate that the variants residing around the γ-secretase site may only affect the production of CICD, but have no effect on the production of ectodomain. Patients with such mutations may only have ASD, in contrast, the patients with *CNTNAP2* gene deletion have both ASD and epilepsy.

CASK belongs to the family of membrane-associated guanylate kinase (MAGUK) proteins. In general, MAGUK proteins target to neuronal synapses and affect the trafficking, targeting, and signaling of ion channels.^53^ Cao et al. demonstrated that RNA interference of CASK in the mPFC leads to impaired social memory in mice,^54^ implicating an important role for CASK in the etiology of ASD.

Recently, Gao et al. demonstrated that CNTNAP2 regulates CASK recruitment to the plasma membrane and stability through the PDZ-binding motif at the C-terminus of CNTNAP2.^50^ However, CASK not only involves in the targeting of synaptic protein but also contributes to the gene expression regulation.^48^ CASK interacts with Tbr-1 and CINAP to regulate the expression of genes, such as *Reelin* and NMDAR subunit 2b (*NR2b*), which may underlie its role in diseases such as ASD. However, it is still unknown how the nuclear-cytoplasmic distribution of CASK is regulated. In our study, we demonstrated that proteolytic cleavage production of CNTNAP2, CICD, promoted the nuclear entry of CASK protein, to regulate the transcription of downstream genes such as *Necdin*. The CICD-CASK interaction is important for the nuclear entry of CASK as CICDΔPDZ, lacking the C-terminal CASK-interactive domain, failed to change the nuclear-cytoplasmic distribution of CASK and the *Necdin* expression. Intriguingly, CASK also interacts with a variety of type I transmembrane proteins, and some of them, such as neurexin, are also involved in the etiology of ASD.^51^ It will be warranted to investigate if the CNTNAP2-CASK-Necdin signaling pathway uncovered in this study is also applicable to the etiology of ASD caused by mutations in other CASK-interacting type I transmembrane proteins. Nonetheless, we cannot totally exclude the possibility that CNTNAP2 regulates *Necdin* expression through other pathways.

Prader-Willi syndrome (PWS) results from the no expression of paternally inherited genes in chromosome 15q11-q13, which is a maternally imprinted region.^55^ PWS is characterized by hyperphagia, repetitive and compulsive behaviors, and cognitive impairment.^56^ Approximately 27% of PWS cases meet ASD diagnostic criteria; however, the underlying mechanism is still elusive.

*Necdin*, one of the genes located in the PWS region, is expressed in all postmitotic neurons and their precursor cells during neuronal development.^57^ Necdin protein plays an important role in brain development, such as proliferation of neural stem cells, maintenance of neuronal survival, neuronal migration and axon growth.^58–61^ Here, we demonstrated that *Necdin*-deficient mice exhibited social deficit, whereas *Necdin* rescued the social deficit in *Cntnap2*^*−/−*^ mice. It has been reported that *Necdin*-deficient mice showed a reduction in oxytocin-producing neurons and alterations of perinatal serotonergic metabolism and development,^62–64^ which may partly underlie the ASD-related behavioral abnormality. Intriguingly, an excess amount of *Necdin* was also reported to induce ASD-related behaviors, which is probably caused by the cortical excitatory-inhibitory imbalance.^46^

*Necdin*, as one of CICD-CASK-regulated genes, can only rescue the social deficit in *Cntnap2*^*−/−*^ mice yet has no effect on the repetitive behavior, indicating the two core deficits in ASD are controlled by different factors/pathways. Indeed, social deficit in *Cntnap2*^*−/−*^ mice has been rescued by certain manipulations, such as suppression of mTOR pathway, administration of oxytocin, or modulation of excitation/inhibition balance in the mPFC. However, these manipulations do not affect the repetitive behaviors in *Cntnap2*^*−/−*^ mice.^32,65,66^ Conversely, risperidone, the first FDA-approved drug for ASD, alleviates hyperactivity and rigid repetitive behaviors in *Cntnap2*^*−/−*^ mice, but does not alter social deficit.^28^ Nevertheless, we found that CICD was able to fully normalize social deficit as well as repetitive behaviors in *Cntnap2*^*−/−*^ mice. Therefore, compounds mimicking CICD may be a novel therapeutic strategy for the core deficits of ASD. And future studies are warranted to further identify the CICD-regulated genes involved in repetitive behaviors.

## Materials and methods

### Mice

*Cntnap2*^*+/-*^ mice at the C57BL/6 J background were acquired from the Jackson Laboratory (#017482). Inter-crossing between *Cntnap2*^*+/-*^ mice was used to produce *Cntnap2*^*−/−*^ mice and wild-type (WT) mice. The genotyping was performed by PCR as previously described.^32^ The generation of *Necdin*-deficient mice at the C57BL/6 J background was described previously.^47^ After weaning, mice of same gender and genotypes were group-housed with 3–5 mice per cage under controlled conditions (temperature, 20 ± 2 °C; relative humidity, 50–60%; 12 h light/12 h dark cycle) and had *ad-lib* access to food and water. All procedures regarding the care and use of animals were approved by the Ethics Committee of School of Life Sciences, Central South University of China. All methods were performed in accordance with approved guidelines.

### Cell culture and transfection

HEK293T cells (ATCC) were maintained in Dulbecco’s modified Eagle’s medium (DMEM, Gibco) supplemented with 10% fetal bovine serum (FBS, Gibco). N2a cells (ATCC) were cultured in DMEM medium supplemented with 10% FBS, and mixed with OptiMem (Gibco) at volume ratio in 1:1. NN cells were maintained in DMEM supplemented with 15% FBS, 1% non-essential amino acid (NEAA, Invitrogen), 0.05‰ LIF (50 U/mL ESGRO Leukemia Inhibitory Factor, Millipore) and another 4 ul β-mercaptan ethanol (Sigma) per 500 mL right before use. All cells were authenticated and tested for mycoplasma contamination, and were maintained at 37 °C in an incubator containing 5% CO2. Plasmid and siRNAs were transfected into cells using Lipofectamine 2000 Reagent (Invitrogen) according to the manufacturer’s instructions.

### Affinity purification of CICD

HEK293T cells were transfected with pRK5-HA-CNTNAP2-GFP-Flag plasmid, and treated with 5 µM MG132 (EMD) overnight at 48 h after transfection. The cell lysates were collected and pre-cleared with 200 µL of Sepharose 4B (Sigma) for 1 h at 4 °C. After centrifugation at top speed for 15 min, the supernatant was transferred into a fresh tube and incubated overnight with 20 µL anti-Flag M2 affinity gel (Sigma). The beads were harvested by centrifugation, washed extensively with lysis buffer at 4 °C, and boiled with 30 µL 1Xloading buffer. After centrifugation at top speed for 10 min, the supernatant was collected for SDS-PAGE.

### Quantitative RT-PCR

Cells or tissues were extracted using TRIzol^®^ reagent (Life technologies, USA) according to the manufacturer’s instruction. 2 μg of total RNA were reverse-transcribed using the RevertAid First Strand cDNA Synthesis Kit (Thermo Fisher, USA; K1622). The mRNA levels (20 ng of total cDNA equivalents) were examined with qPCR using Fast SYBR™ Green Master Mix (Thermo Fisher, USA; 4385612) according to the manufacturer’s instructions by a C1000 touch Thermal Cycler. Primers used for qPCR were shown as follow: Gapdh forward primer: AGGTCGGTGTGAACGGATTTG**;** Gapdh reverse primer: TGTAGACCATGTAGTTGAGGTC A**;** Necdin forward primer: GAGGTCCCCGACTGTGAGAT; Necdin reverse primer: TGCAGG ATTTTAGGGTCAACATC**;** Cntnap2 forward primer: CCTTGGCACCTAGATCACTTG**;** Cntnap2 reverse primer: CCCCTCCAATGATAGCTGAGTTT**;** Cask forward primer: TGGAAG CTCTACGCTACTGC**;** Cask reverse primer: GTTTAACAGGTGCCGAGTTTTC. Primers used for ChIP-qPCR were shown as following: P1 forward primer: CAACACGCATGCGCAATATC; P1 reverse primer: GATGCGGCTTGGAGCTCTT; P2 forward primer: CTAGTTCTGTGCCATACAGGAGAC; P2 reverse primer: GCGGGGCTGATGCGATATT; P3 forward primer: ACTCATCATCATCATAAGGTACAGC; P3 reverse primer: TGTGAAGGTCCTGGAGAAAGAC; P4 forward primer: ACATGGATTTATCTCCAGTGTCTG; P4 reverse primer: GGAAAGCTGTACCTTATGATGATG; P5 forward primer: GATCATTTTCCACTAGAATCTTAACGGAAG; P5 reverse primer: GCCCCACATGAAAATGAGGGATAT.

### Nuclear and cytoplasmic protein isolation

N2a cells on 6 cm plates were transfected with 1 μg expression construct (pRK5, pRK5-HA-CNTNAP2-Flag, pRK5-CICD or pRK5-CICDΔPDZ respectively) or 10 μL siRNAs (20 μM) using Lipofectamine 2000 Reagent (Invitrogen) according to the manufacturer’s instructions. At 48 h after transfection, cells were washed with cold 1XDPBS (Gibco) and harvested using a cell scraper. Cells were transferred to a 1.5 mL Eppendorf tube and spun at 3000 g for 5 min at 4 °C. After removal of the supernatant, cells were re-suspended in 200 μL cytoplasmic extraction reagent (CER, 10 mM HEPES or Tris PH7.5, 40 mM KCl, 2 mM MgCl2, 10% glycerol and 1×Protein Inhibitor Cocktail) and incubated for 10 min on ice. Further, cells were blown softly about 25–40 times with a 1 mL syringe until the suspension was thick. The suspension was centrifuged at 4,000 *g* for 5 min at 4 °C and the supernatant (cytoplasmic fraction) was transferred to a new Eppendorf tube. The pellet was washed twice with 1XDPBS. Thereafter, the pellet was re-suspended in 3 mL 0.25 M sucrose solution (0.25 M sucrose, 1 M MgCl_2,_ 1 M HEPES PH 7.5) and transferred carefully to a 15 mL Falcon tube containing 3 mL 0.35 M sucrose solution (0.35 M sucrose, 1 M MgCl2, 1 M HEPES PH 7.5). Then, the density gradient centrifugation was done at 1430 *g* for 5 min at 4 °C. The supernatant was removed and the pellet (nuclear fraction) was re-suspended in 200 μL nuclear extraction reagent (NER, 10 mM HEPES or Tris PH7.5, 500 mM NaCl, 1%Triton-X100, 10% glycerol and 1×Protein Inhibitor Cocktail). The suspension was ultrasonicated on ice for 20 s and centrifugation for 15 min at 13,000 *g*. Then, the samples were mixed with 2×SDS lysis buffer and boiled for 10 min. After centrifugation for 5 min at 13,000 *g*, the supernatant (nuclear fraction) was transferred to a new Eppendorf tube.

The mPFC was dissected and cut into small pieces. After addition of cytoplasmic extraction reagent (200 μL per 60 mg), tissues were transferred into Dounce Tissue Homogenizer and fully homogenized. The homogenate was transferred to a 1.5 mL Eppendorf tube and spun at 1500 g for 5 min at 4 °C. The supernatant was left as cytoplasmic fraction, whereas the pellet was further processed as above.

### Western blotting

The samples were mixed with 2×SDS lysis buffer (50 mM Tris–HCl at pH 6.8, 2% SDS and 10% glycerol) with 1×Protein Inhibitor Cocktail, boiled at 100 °C for 10 min. The supernatant was obtained by centrifugation at 13,000 *g* for 10 min and the protein concentration was determined using the Pierce^TM^ BCA protein Assay kit (Thermo Fisher). Proteins in lysates were separated by SDS-PAGE, transferred to PVDF membranes, and blocked in 5% skim milk that contained 0.1% Tween 20 at room temperature for 1 h. The membranes were immunoblotted with the corresponding antibodies overnight at 4 °C, and then were washed and incubated with horseradish peroxidase conjugate secondary antibodies at room temperature for 1 h. After washing, the bands were visualized using the Pierce™ ECL Western Blotting Substrate kit (Thermo Fisher) and band intensities were quantified by ImageJ. The antibodies were listed as following: CNTNAP2 (ab33994, Abcam), Necdin (ab18554, Abcam), Flag-tag (14793, CST), HA-tag (3724, CST), GFP (2555, CST), CASK (ab252540, Abcam), GAPDH (97166, CST), Histone H3(9715, CST), PS1 (ab15458, Abcam).

### Chromatin immunoprecipitation (ChIP)

N2a cells were transfected with 3 μg plasmids (pRK5-Myc or pRK5-CASK-Myc) using Lipofectamine 2000 Reagent (Invitrogen) according to the manufacturer’s instructions. At 48 h after transfection, fresh 37% formaldehyde was added to cultured cells to a final concentration of 1%. After incubation at room temperature for 10 min, 1/20 volume of 2.5 M glycine was added to quench formaldehyde. The cells were rinsed twice with cold 1XDPBS (Gibco) and harvested using a silicon scraper. The cells were then transferred to a 1.5 mL Eppendorf tube and spun at 1350 g for 5 min at 4 °C. After removal of the supernatant, cells were re-suspended in 500 μL Lysis Buffer1 (50 mM Hepes-KOH, 140 mM NaCl, 1 mM EDTA, 10% glycerol, 0.5% NP-40, 0.25% Triton X-100) and incubated at 4 °C for 10 min. After spinning at 1,350 g for 5 min at 4 °C, each pellet was resuspended in 500 μL Lysis Buffer 2 (10 mM Tris-HCl, 200 mM NaCl, 1 mM EDTA, 0.5 mM EGTA). After rocking gently at room temperature for 10 min, the nuclei were pelleted in tabletop centrifuge by spinning at 1350 g for 5 minutes at 4 °C. Each pellet was resuspended in each tube in 200 μL Lysis Buffer 3 (10 mM Tris-HCl, 100 mM NaCl, 1 mM EDTA, 0.5 mM EGTA, 0.1% Na-Deoxycholate, 0.5% N-lauroylsarcosine). The suspension was sonicated with a microtip attached to Misonix 3000 sonicator in an ice water bath. 50 µL of 10% Triton X-100, 200 µL Lysis Buffer 3, 5 μL cocktail, and 5 μL anti-Myc (CST, 2276 S) were added to each sample and incubated overnight at 4 °C. 15 μL beads (Thermo Fisher) was added and incubated at 4 °C for 4 h. The beads were washed with 1 mL Wash Buffer (50 mM Hepes-KOH, 500 mM LiCl, 1 mM EDTA, 1% NP-40, 10% Na-Deoxycholate) and eluted with 100 µL elution buffer (50 mM Tris-HCl, 10 mM EDTA, 1% SDS) at 65 °C with agitation for 2-3 h. 3-4 µL of 25 mg/mL RNaseA was added into each sample and incubated at 65 °C with agitation for 4-5 h, then 2 µL of 10 mg/mL proteinase K was added and incubated at 55 °C with agitation for 4-5 h. 200 µL AMPure XP Beads (Beckman, A63881) was used to extract DNA for qPCR assay.

### Luciferase reporter assays

HEK293T cells were plated on 24-well plates and transfected with 20 ng, 40 ng, 80 ng of pRK5-CASK, 5 ng pGL3-Necdin promoter-Luc and 10 ng of pCMV-β-gal. At 48 h after transfection, cells were washed twice with PBS and lysed with Reporter Lysis Buffer (Promega). The extracts were assayed for luciferase activity with the Luciferase Assay System (Promega). Luciferase activities were determined using SIRIUS luminometer (Berthold Detection Systems GmbH).

### Three-chamber social interaction test

Male mice at the age of 6–8 weeks were used. Mice were habituated to the experimenter for at least 3 days prior to the start of the behavioral experiments. Animals were allowed to acclimate to the behavioral testing room for at least 1 h before the first trial begins. The apparatus was a clear Plexiglas box divided into three interconnected equally-sized interconnected chambers (left, center, right), mouse was able to access each chamber from the center through the retractable doorways. There are two phases in the three-chamber social interaction test: habituation and sociability.^67^

At habituation phase, we placed two plexiglas cages in left and right chambers, the position was counter balanced in order to avoid bias, and two identical objects were placed inside. A subject mouse was introduced into the central chamber, and was allowed to explore three chambers for ten min. During habituation, the subject’s position was tracked continuously with an automated tracking software.

At sociability phase, an age- and sex-matched stranger mouse was enclosed in a plexiglas cage so that the social interaction was only initiated by the test mouse. To measure the sociability, we placed the subject mouse into the central chamber, and allowed it to explore the three-chamber apparatus for ten minutes. The behaviors were recorded and Anilab Software (Anilab) was used to score the time that the subject mouse spent on sniffing or climbing upon each plexiglas cage. We used the following formulate to calculate the preference index: Preference index =Time exploring (stranger mouse – object) / Time exploring (stranger mouse + object).

### Reciprocal interaction test

We placed a male mouse at the age of 6–8 weeks in a cage and allow it to habituate for 10 min. Then, a novel conspecific matched mouse according to genotype, age, sex, and/or treatment was placed in a neutral arena. The time spent in social interaction of both animals was measured by two independent observers blind to the genotypes, including touching, close following, nose-to-anus sniffing, nose-to-nose sniffing, grooming and/or crawling over/under each other.^68^

### Grooming test

We placed a male mice at the age of 6–8 weeks in a Plexiglas column (20 cm diameter). The behaviors were recorded for 10 min after 10 min acclimation. The time spent on grooming themselves was measured by a researcher who is blind to the genotypes.

### Stereotaxic injection

Adeno-associated viruses carrying genes for EGFP (rAAV-EF1a-EGFP-WPRE-hGH pA, AAV-EGFP), CICD (rAAV-EF1a-CICD-P2A-EGFP-WPRE-hGH pA, AAV-CICD) or Necdin (rAAV-EF1a-Necdin-P2A-EGFP-WPRE-hGH pA, AAV-Necdin) was used. AAV-EGFP (AAV2/9, 2.46 × 10^12^ genomic copies per mL), AAV-CICD (AAV2/9, 5.55 × 10^12^ genomic copies per mL), AAV-Necdin (AAV2/9, 5.61 × 10^12^ genomic copies per mL) were made by BrainVTA (Wuhan). WT or *Cntnap2*^*−/−*^ mice (4 weeks old), randomly allocated for different virus injection, were anesthetized with isoflurane (induction 4%, maintenance 1%) and placed in a stereotaxic frame (RWD). The skull was exposed under antiseptic conditions and a small craniotomy was made with a thin drill over prefrontal cortex (typical coordinates: 1.98 mm anterior to Bregma; ±0.4 mm lateral to the midline). AAV-EGFP, AAV-CICD or AAV-Necdin were injected using a glass micropipette (tip diameter ~15 µm) attached to a Nanoliter 1000 pressure injection apparatus. Over a 10 min period, 0.2 µL of virus was injected at a depth of 1.65 mm from the Bregma. The pipette remained for 10 min at the end of infusion to allow virus diffusion. When pulled out, pipette remained for 1 min per 0.05 mm lift to localize virus. The mice were sutured and placed on a hot blanket until they woke up. Behavior experiments were conducted at 3 weeks after virus injection.

### Statistical analysis

Statistical analyses were performed using GraphPad Prism 9.3.0. Except behavior tests, all experiments were repeated at least three times. Data were presented as mean ± SEM and the exact statistical tests each experiment were stated in the Figure legend.

## Supplementary information

Supplementary Methods and Figures

## Data Availability

The data that support the findings of this study are available from the corresponding author upon request.
