# Supplementary material for: CNTNAP2 intracellular domain (CICD) generated by γ-secretase cleavage improves autism-related behaviors
Source: Signal Transduct Target Ther. 2023 Jun 5;8:219. doi: 10.1038/s41392-023-01431-6 (PMC10239753; doi:10.1038/s41392-023-01431-6)
Supplement: Supplementary file 1 — Supplementary Methods and Figures [file 41392_2023_1431_MOESM1_ESM.pdf]

Supplementary Materials for

CNTNAP2 intracellular domain (CICD) generated by γ-secretase cleavage improves autism-related behaviors

Jing Zhang†, Fang Cai†, Renbin Lu, Xiaoliang Xing, Lu Xu, Kunyang Wu, Zishan Gong, Qing Zhang, Yun Zhang, Mengen Xing, Weihong Song*, Jia-Da Li*

Correspondence to: Weihong Song (weihong@wmu.edu.cn)

Jia-da Li (lijiada@sklmg.edu.cn)

This PDF file includes:

1. Materials and Methods
2. Figures S1 to S10

Materials and Methods

Sequences for RNA interference

The siRNA sequence of Negative Control was 5'-UUCUCCGAACGUGUCACGUTT-3'. The sequences for the sense strand of *Cntnap2* siRNAs and *Cask* siRNAs were as follow: siRNA-*Cntnap2*-1: 5'-GCACACGCUUACACUAUCATT, siRNA-*Cntnap2*-2: 5'-CUGCCAUCAUGAACAACGATT, and siRNA-Cask-1: 5'-GGAAGAGAUUCACGCACUUT, siRNA-Cask-2: 5'-GGAAGAAGCUGUCGAGCUUTT. The target sequences of Cask shRNA were as following: shRNA-1: CGGCGATGTATCAACAGAGAA; shRNA-1: GCGAGGGAGTATTACCTTCAA.

Primary neuronal culture

Primary cortical and hippocampal cultures were prepared from brains of embryonic day (E) 19-20 C57BL/6J mice. The mouse cortices and the hippocampus were obtained in ice HBSS (Gibco, no phenol red) under a dissection microscope. The tissues were cut and digested with papain (mixed well with DMEM, 1:20) at 37°C with gentle rotation for 30-45 minutes. The digestion solution was removed and cells were dissociated by pipetting with MEM + GlutaMAXTM (containing 10% FBS, Gibco). The suspension was transferred into a cell strainer. Dissociated neurons were plated at 3 x105 cells per cm2 on 24-well plates coated with poly-l-ornithine (Sigma). After 3-4 hours, the cultured medium was replaced with Neurobasal medium (Invitrogen) supplemented with 2 mM L-Glutamine, 0.25g/mL plasmocin and B-27 supplement (Invitrogen). On the following day, cultures were maintained at 37°C in a 5% CO2 incubator and half of the medium was changed every 3 or 4 days before use. Transfection or other treatment were done at 5 days in vitro (DIV5), and neurons were lysed in 2×SDS lysis buffer at DIV 14 or 5 days after lentivirus infection.

Mass spectrometry (MS)

The affinity-purified samples were separated by 10% SDS-PAGE. The gel was incubated in 0.1% (w/v) Coomassie brilliant blue R-250 (supplemented with 40% methanol and 10% acetic acid) for 1h, and washed in de-staining solution (40% methanol and 10% acetic acid) overnight. The target bands in gel were excised, reduced with dithiothreitol, and alkylated with iodoacetamide. The samples were then digested with trypsin at volume ratio in 1:50 (sample: enzyme). Each of the digested protein samples were extracted, and the peptides were desalted using STAGE-tips. Briefly, the peptides were loaded onto C18 material immobilized in a pipette tip, then washed with aqueous 0.1% formic acid, and eluted with 80% acetonitrile (including 0.1% formic acid). The resultant eluent was dried for subsequent resuspension in MS loading buffer. The resuspended peptide mixtures were then loaded onto a nanoflow-LC-MS/MS system (Bruker Impact II Q-Tof, with Proxeon EasyLC system, featuring in-house packed 400mm × 50um integrated emitter columns) and run with 90 mins H_2O: ACN gradients. The mass spectrometer was operated in data-dependant MS/MS mode, with up to 15 MS/MS scans triggered from every MS scan. The data was searched with the Byonic search engine (v3.4.0-ProteinMetrics Inc.) against the human proteome database appended with the proposed sequence for the custom protein. Search parameters were set to: Ion mass tolerances 10ppm and 40ppm precursor and fragment ion mass tolerances, respectively. Semi-specific tryptic peptide specificity. 1% protein FDR and peptide Byonic score cut-off of 200. Modifications were fixed carbamidomethyl C, and variable oxidized M and deamidated N. In addition, the data was manually searched for the proposed novel N-terminal peptides, first by extracting the ion chromatograms of the most likely charge states, and verifying that they overlap during chromatographic elution, and then manually validating the sequence of the peptide by MS/MS.

N-terminal sequencing

The affinity-purified samples were separated by 10% SDS-PAGE, and transferred to the PVDF membrane. The PVDF was incubated in 0.1% (w/v) Coomassie brilliant blue R-250 (supplemented with 40% methanol and 10% acetic acid) for 5 minutes and washed in de-staining solution (40% methanol and 10% acetic acid) for 15 minutes. The PVDF membranes were rinsed in 90% methanol (added 5% acetic acid) for 45 sec, followed by distilled water overnight (changing the water several times). The PVDF membranes were dried between Whatman No. 1 filter papers for N-terminal sequencing. The target bands were washed 6 times with deionized water and loaded onto the instrument (Shimadzu PPSQ-53A) for sequence analysis using Edman degradation.

Immunofluorescent staining

After perfusion intracardially with 4% paraformaldehyde, the brains were taken from the mice and post-fixed and cryoprotected overnight in 25% sucrose. Twenty-micron-thick brain sections including the mPFC region were obtained and incubated with blocking buffer (10% FBS, 5%BSA, 0.3% TritonX-100, 0.01% NaN_3) at room temperature for one hour. The slices were then incubated overnight with primary antibodies to NeuN (1:300, Abcam), PV (1:500, Sigma), GFAP (1:500, Millipore), Iba1 (1:1000, Wako), respectively. The slices were then incubated with Alexa-conjugated secondary antibodies in the dark. After staining the nuclei with DAPI, slices were imaged by using a confocal microscope (TCS SP5; Leica).

To measure the subcellular localization, N2a cells plated on coverslips were co-transfected with pRK5, pRK5-CICD, pRK5-CICDΔPDZ or pRK5-3A-CICD and pRK5-CASK-Myc, respectively. At twenty-four hours after transfection, cells were treated with 20μM MG132. At forty-eight hours after transfection, cells were fixed in 4% paraformaldehyde for 15 min at room

temperature. Cells were treated with 0.2% TritonX-100 for 30 min at room temperature for permeabilization and incubated with 5% bovine serum albumin for 1 hour. Mouse anti-Flag M2 (1:400 dilution; Millipore Sigma) and rabbit anti-Myc (1:400 dilution; CST) was added and incubated at 4 ℃ overnight. After PBS wash, fluorescence-labeled secondary anti-mouse or rabbit antibodies (1:400 dilution; Thermo Fisher Scientific) were added and incubated for 1 hour in the dark at room temperature. After staining the nuclei with DAPI, slices were imaged by using a confocal microscope (TCS SP5; Leica).

Lentivirus package and infection

The shRNAs targeting Cask were inserted into pLKO.1-TRC Cloning Vector (Addgene). HEK293T cells were transfected with 44μg constructs (pLKO.1-TRC, pLKO.1-TRC-shRNA1 or pLKO.1-TRC-shRNA2), 30μg psPAX2, 10μg pMD2G and 10μg pAdvantage using a calcium phosphate transfection method. At 72 hours after transfection, the culture medium was harvested in a conical tube and centrifuged at 4000 rpm for 10 min at 4 ℃. The supernatant was centrifuged at 25000 rpm for 90 min at 4°C, and the pellet was incubated with 100µL HBSS (Gibco, no phenol red) overnight. The resuspension solution (lentivirus) was divided into 1.5 mL Eppendorf tubes and stored in -80 ℃ until use. Each 24-well of primary cortical neuron was transfected with 0.5-1µL lentivirus, and protein was harvested after 5 days for immunoblotting.

Co-immunoprecipitation assay

N2a cells were plated on 6-cm dishes and co-transfected with pRK5, pRK5-CICD or pRK5-CICDΔPDZ, respectively. At twenty-four hours after transfection, cells were treated with 20μM MG132. At forty-eight hours after transfection, we harvested cells with lysis buffer (150 mM NaCl, 1% NP-40, 2 mM EDTA, 50 mM Tris, pH 8.0, and protease inhibitor cocktail). About 1 mg of whole cell lysate was mixed with 1μg of the indicated antibody under constant agitation overnight

at 4°C. Then, we added 30μL protein G agarose bead slurry (Sigma) to pull down the immunocomplexes. After centrifugation, the beads were washed extensively with lysis buffer for 3-5 times, and boiled with 2× SDS loading buffer. The protein samples were then subjected to Western blot analysis with the appropriate antibody.

Supplemental Figures

Figure S1

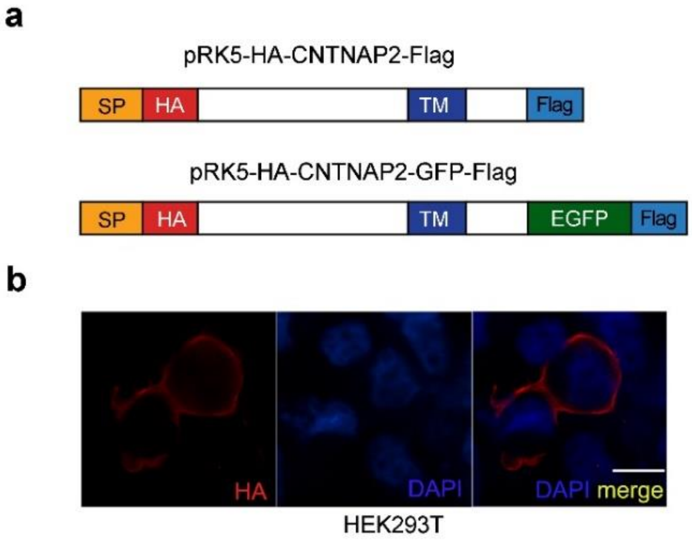

Figure. S1. CNTNAP2 was expressed on the cell surface. (a) Schematic illustration of HA-CNTNAP2-Flag (upper) and HA-CNTNAP2-EGFP-Flag (bottom). SP, signal peptide; TM, transmembrane domain. EGFP, enhanced green fluorescence protein. **(b)** HA-CNTNAP2-Flag was detected on the cell surface. HEK293T cells transfected with pRK5-HA-CNTNAP2-Flag were measured with immunofluorescence without permeabilization using an HA antibody. Scale bar, 5 μm.

Figure S2

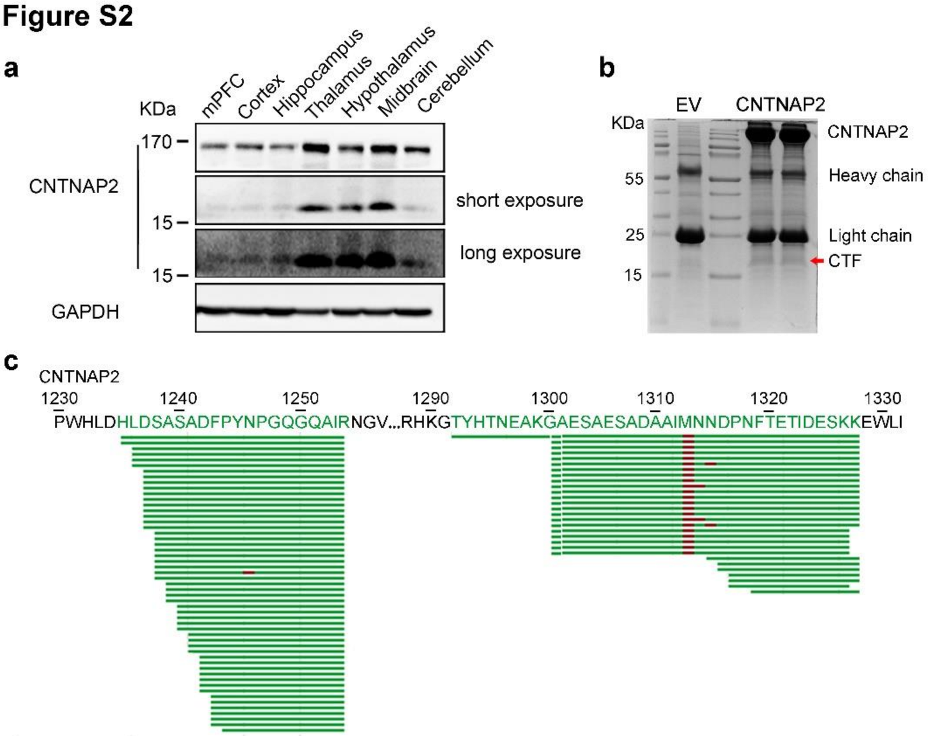

Figure S2. Characterization of the ~20KDa CTF fragment. (a) Identification of ~20KDa CTF fragment in different brain regions by using a CNTNAP2 antibody. **(b)** Affinity-purification of CNTNAP2 and its proteolytic cleavage products. HEK293T cells were transfected with empty vector (EV) or pRK5-HA-CNTNAP2-Flag (CNTNAP2). Cell lysates were immunoprecipitated with a Flag antibody, and separated with SDS-PAGE. The gel was stained with Coomassie blue and the red arrow indicates the ~20KDa fragments corresponding to CTF. **(c)** The CTF indicated with a red arrow in (b) was subjected to mass spectrometry (MS) analysis, and the peptides corresponding to the C-terminus of CNTNAP2 were shown as green lines. Each green line represented one hit in the MS analysis.

Figure S3

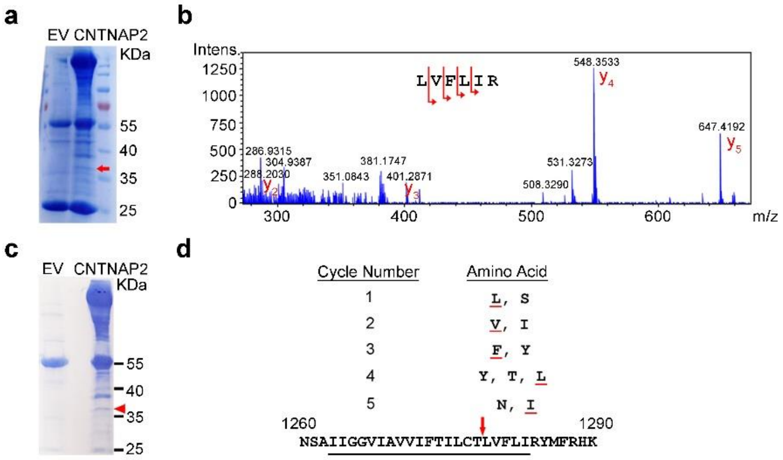

Figure S3. Identification of the N-terminal sequences of CICD. (a) HEK293T cells were transfected with empty vector (EV) or pRK5-HA-CNTNAP2-GFP-Flag (CNTNAP2). Cell lysates were affinity-purified with anti-Flag affinity gel, and separated with SDS-PAGE. The gel was stained with Coomassie blue and the red arrow indicates the ~35KDa fragments corresponding to CICD. **(b)** MS analysis of the ~35KDa fragments marked in (**a**). **(c)** The protein on the gel in (**a**) was transferred onto PVDF membrane and stained with Coomassie blue. The red arrow indicates the ~35KDa fragments corresponding to CICD. **(d)** N-terminal sequencing result of the ~35KDa fragments marked in (**c**). The lanky red arrow indicates the start of the N-terminus of CICD.

Figure S4

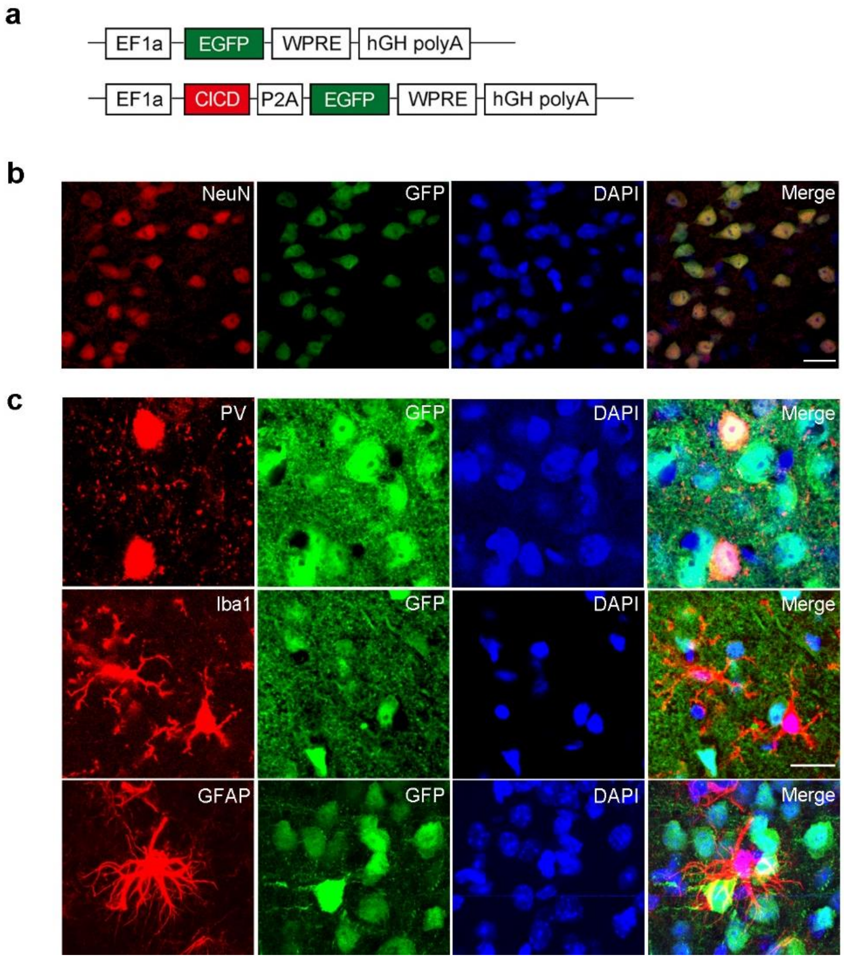

Figure S4. Infection of AAV into neurons but not astrocytes and microglia. (a) Schematic illustration of AAV-EGFP (Top) and AAV-CICD (Bottom). CICD and EGFP were expressed separately due to the insertion of P2A between them. P2A, self-cleaving 2A peptide from Porcine teschovirus 1. **(b)** EGFP colocalized with neuron marker NeuN. The fluorescence signal of EGFP was shown in green, whereas NeuN was immune-stained in red with corresponding antibodies. Scale bar, 20 μm. **(c)** EGFP colocalized with inhibitory neuron marker PV, but not microglia marker Iba1 and astrocyte marker GFAP. The fluorescence signal of EGFP was shown in green, whereas PV, Iba1 and GFAP were immune-stained in red with corresponding antibodies. Scale bar, 20 μm.

Figure S5

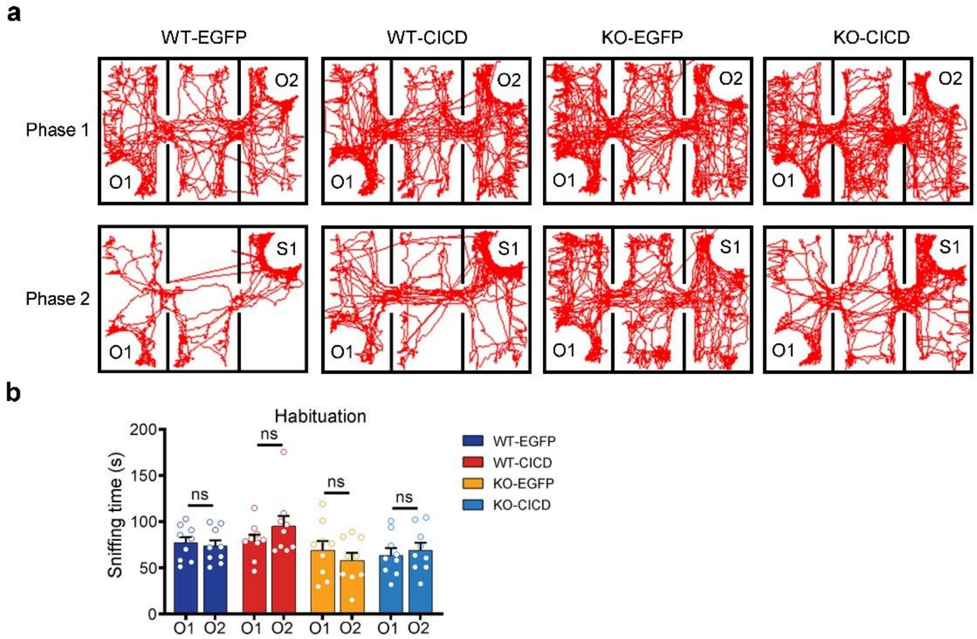

Figure S5. CICD rescues autism-related behaviors in *Cntnap2*$^{-/-}$ mice. (a) The representative tracing images of animal movement during the three-chamber assay. (b) There was no bias in test mice at habituation phase. The sniffing time of test mice spent on two identical objects, object 1 (O1) or object 2 (O2), during the habituation phase in a three-chamber test. Statistics data were presented as mean ± SEM, n=9; ns, no significance, unpaired *t*-test.

Figure S6

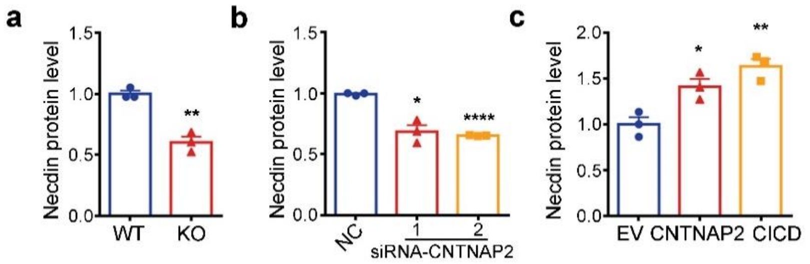

Figure S6. CNTNAP2 and CICD positively regulated the transcription of *Necdin.* (a) The statistics data for the relative Necdin protein levels in Fig. 3c. Necdin protein level was significantly down-regulated in the mPFC of *Cntnap2*$^{-/-}$ mice. Data were presented as mean ± SEM, n=3; **p<0.01, unpaired t-test. **(b)** The statistics data for the relative Necdin protein levels in Fig. 3e. RNA interference of *Cntnap2* in N2a cells led to reduction of protein levels of Necdin. Data were presented as mean ± SEM, n=3; *p<0.05, ****p<0.0001, unpaired t-test. **(c)** The statistics data for the relative Necdin protein levels in Fig. 3g. Overexpression of CNTNAP2 or CICD in N2A cells significantly upregulated the protein levels of endogenous Necdin. Data were presented as mean ± SEM, n=3; *p<0.05, **p < 0.01, unpaired t-test.

Figure S7

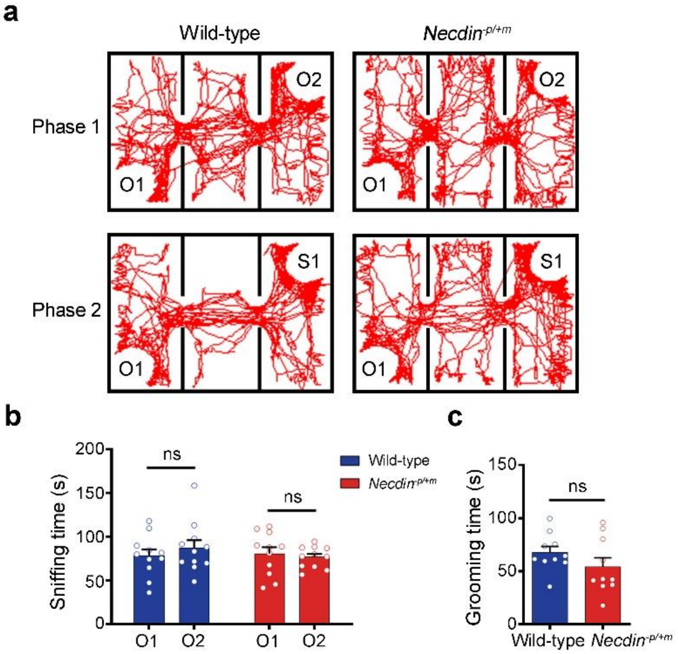

Figure S7. The behavioral tests of *Necdin*$^{-p/+m}$ mice. (a) The representative tracing images of animal movement during the three-chamber assay. **(b)** The sniffing time of test mice spent on two identical objects, object 1 (O1) or object 2 (O2), during the habituation phase in a three-chamber test. **(c)** The time of test mice spent on self-grooming. All data were presented as mean ± SEM, n=10 mice/genotype; ns, no significance, unpaired *t*-test.

Figure S8

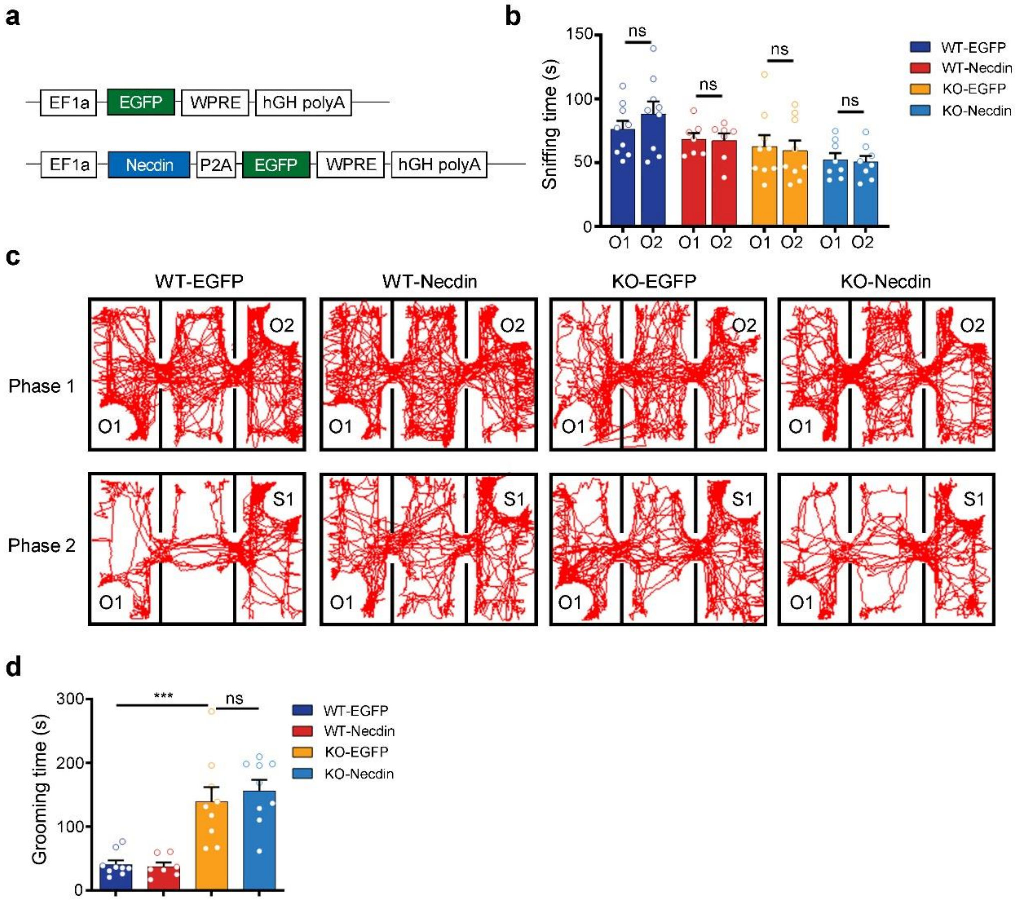

Figure S8. The behavioral tests of *Cntnap2*$^{-/-}$ mice after virally delivery of Necdin to the mPFC. (a) Schematic illustration of the control AAV-EGFP (Top) and the AAV overexpressing Necdin (Bottom). **(b)** The sniffing time of test mice spent on two identical objects, object 1 (O1) or object 2 (O2), during the habituation phase in a three-chamber test. **(c)** The representative tracing images of animal movement during the three-chamber assay. **(d)** The time of test mice spent on self-grooming. Virally delivery of Necdin to the mPFC had no effect on the repetitive behavior. All data were presented as mean ± SEM, n=9; ns, no significance, ***p<0.001, unpaired t-test.

Figure S9

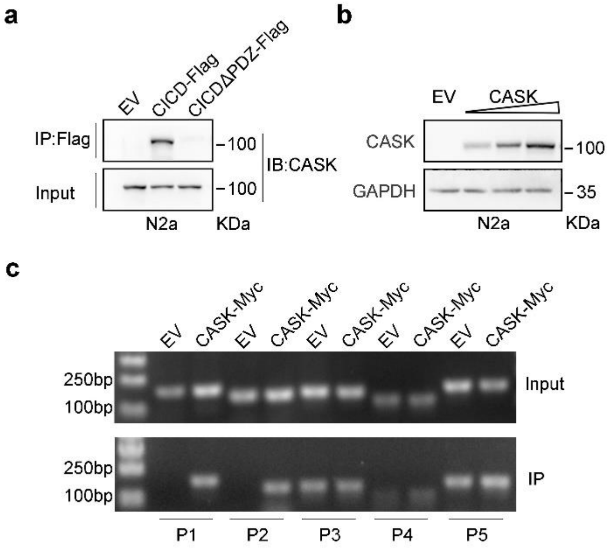

Figure S9. CASK regulates the transcription of *Necdin*. (a) CICD interacted with CASK through the C-terminal PDZ-binding motif. N2a cells were transfected with a CASK-expressing plasmid together with an empty vector (EV), a CICD-Flag-expressing plasmid, or a CICDΔPDZ-Flag-expressing plasmid. Cell lysates were subjected to co-immunoprecipitation (co-IP) assay with an antibody against Flag, and the immune complex was blotted with the antibody against CASK. **(b)** The protein levels of CASK in N2a cells transfected with 0, 20, 40, 80ng of CASK-expressing plasmid as detected with Western blot (related to Figure 5g). The CASK protein levels proportionally increased with increasing concentrations of plasmid. EV, empty vector. **(c)** The electrophoresis gel images of ChIP-qPCR products (related to Figure 5h).

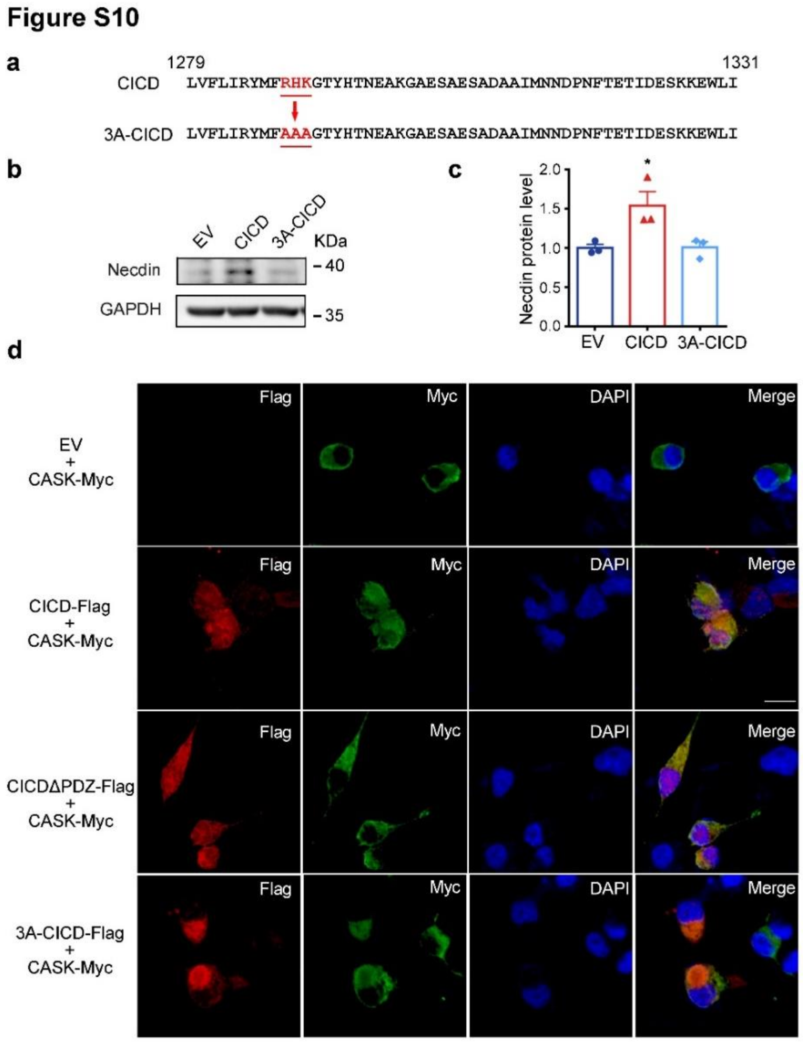

Figure S10. The triplet of basic amino acids (RHK) at the N-terminus of CICD is critical for promoting the nuclear entry of CASK. (a) The sequences of CICD. The triplet of basic amino acids (RHK) was highlighted and mutated to AAA, generating 3A-CICD. **(b, c)** Overexpression of 3A-CICD in N2a cells failed to up-regulate Necdin protein level. Data presented in (**c**) were mean ± SEM, n=3; **p<0.01, unpaired *t*-test. **(d)** Immunofluorescence staining of N2a cells transfected with CASK-Myc-expressing plasmid together with an empty vector (EV), a CICD-Flag-expressing plasmid, a CICDΔPDZ-Flag-expressing plasmid, or a 3A-CICD-Flag-expressing plasmid. DAPI was used to visualize the nuclei. Scale bar: 10μm.
